# Increased integrated testing for HIV, hepatitis C and sexually transmitted infections in health care facilities: results from the INTEGRATE Joint Action pilots in Lithuania, Romania and Spain

**DOI:** 10.1186/s12879-021-06537-2

**Published:** 2021-09-13

**Authors:** R. Matulionytė, M. L. Jakobsen, V. I. Grecu, J. Grigaitiene, T. Raudonis, L. Stoniene, M. Olteanu, L. de la Mora, D. Raben, A. K. Sullivan

**Affiliations:** 1 Department of Infectious Diseases and Dermatovenerology, Faculty of Medicine, Vilnius University, Vilnius University Hospital Santaros Klinikos, Vilnius, Lithuania; 2 Department of Infectious Diseases, CHIP, Rigshospitalet, Copenhagen, Denmark; 3 Victor Babes’ Clinical Hospital of Infectious Diseases and Pneumophtisiology, Craiova, Romania; 4 Republican Centre for Addictive Disordes, Giedraičių g. 8, LT–03147 Lithuania, Kaunas Lithuania; 5 HIV Clinic, Hospital Clinic Barcelona, Barcelona, Spain; 6 Chelsea & Westminster Hospital NHS Foundation Trust, London, UK

**Keywords:** HIV, Viral hepatitis, Integrated testing, Sexually transmitted infections

## Abstract

**Background:**

Indicator condition guided HIV testing is a proven effective strategy for increasing HIV diagnosis in health care facilities. As part of the INTEGRATE Joint Action, we conducted four pilot studies, aiming to increase integrated testing for HIV/HCV/HBV and sexually transmitted infections, by introducing and expanding existing indicator condition guided HIV testing methods.

**Methods:**

Pilot interventions included combined HIV/HCV testing in a dermatovenerology clinic and a clinic for addictive disorders in Lithuania; Increasing HIV testing rates in a tuberculosis clinic in Romania by introducing a patient information leaflet and offering testing for HIV/HCV/sexually transmitted infections to chemsex-users in Barcelona. Methods for implementing indicator condition guided HIV testing were adapted to include integrated testing. Testing data were collected retrospectively and prospectively. Staff were trained in all settings, Plan-do-study-act cycles frequently performed and barriers to implementation reported.

**Results:**

In established indicator conditions, HIV absolute testing rates increased from 10.6 to 71% in the dermatovenerology clinic over an 18 months period. HIV testing rates improved from 67.4% at baseline to 94% in the tuberculosis clinic. HCV testing was added to all individuals in the dermatovenerology clinic, eight patients of 1701 tested positive (0.47%). HBV testing was added to individuals with sexually transmitted infections with a 0.44% positivity rate (2/452 tested positive). The Indicator condition guided HIV testing strategy was expanded to offer HIV/HCV testing to people with alcohol dependency and chemsex-users. 52% of chemsex-users tested positive for ≥ 1 sexually transmitted infection and among people with alcohol dependency 0.3 and 3.7% tested positive for HIV and HCV respectively.

**Conclusions:**

The four pilot studies successfully increased integrated testing in health care settings, by introducing testing for HBV/HCV and sexually transmitted infections along with HIV testing for established indicator conditions and expanding the strategy to include new indicators; alcohol dependency and chemsex. HCV testing of individuals with alcohol abuse showed high positivity rates and calls for further implementation studies. Methods used for implementing indicator condition guided HIV Testing have proven transferable to implementation of integrated testing.

**Supplementary Information:**

The online version contains supplementary material available at 10.1186/s12879-021-06537-2.

## Background

HIV, hepatitis B (HBV) and hepatitis C (HCV) and sexually transmitted infections (STI) share the same modes of transmission and co-infections are common, meaning people at risk of acquiring one infection will often also be at risk of catching one of the other infections. Additionally, a large proportion of people infected, especially with HIV, HBV and HCV, are unaware of their infection [1, 2]—A public health challenge, that continues to fuel onward transmission and jeopardizing individual health.^1^

Indicator condition guided HIV testing (ICT) (routine testing for HIV based on presentation with a condition or disease with a proven HIV prevalence of > 0.1%) [3–6] has proven a feasible, efficient and cost- effective strategy to increase diagnoses of HIV in health care settings, when people present with other health-related issues. ICT is recommended by European HIV testing guidelines and in many national testing guidelines [7, 8]. Additionally, several tools to facilitate the practical implementation of ICT have been developed, tested and proved to be very successful in supporting routine ICT [3, 4, 9]. However, ICT is still not systematically and fully implemented across all relevant sectors and health care settings in Europe, resulting in many missed opportunities for diagnosis. The level of undiagnosed viral hepatitis and STI is generally even higher than HIV [10]—although monitoring of these infections is still insufficient. However, this calls for increased testing for viral hepatitis and STIs, which is also acknowledged in recent European guidelines and policies [7]

This article reports the results of pilot interventions conducted in four different health care settings in Europe as part of the INTEGRATE Joint Action [11] with the aim of expanding existing ICT strategies and adapting tools from an HIV context to an integrated testing approach. This was done firstly by offering a combination of testing for HCV, HBV and STI along with offering HIV testing—integrated testing. Secondly by using the ICT strategy for routine testing to implement integrated testing in other high-risk indicators like alcohol dependency and chemsex. The overall study aim was to increase integrated testing coverage and earlier diagnosis for HIV, HCV, HBV and STIs in health care settings.

## Methods

Four pilots were conducted: in a dermatovenerology clinic at the Vilnius University Hospital in Lithuania, where individuals presenting with HIV indicator conditions (IC) were offered testing for both HIV and HCV, in addition, individuals presenting with an STI were also offered testing for HBV. Also in Lithuania, the five regional clinics for addictive disorders at the Republican Centre for Addictive Disorders (RPLC), Vilnius, Kaunas, Klaipeda, Siauliai and Panevezys, routine offer of combined HIV and HCV testing to people with a history of intravenous drug use (PWID) on opioid substitution therapy (OST) was extended to people with alcohol dependency. In Victor Babes Hospital for infectious diseases and lung infections including tuberculosis (TB) in Craiova, Romania, the aim was to increase HIV testing rates in individuals presenting with TB and reduce the time doctors take to obtain the legally required, informed, signed consent. Finally, an HIV clinic at Hospital Clinic de Barcelona, Spain, established a referral system in collaboration with various health and non-health care facilities (emergency departments; HIV, STIs and PEP units; Psychiatry/addictions services and a local NGO) where individuals reporting a history of chemsex, were referred to the HIV clinic and offered testing for HIV, HCV and STIs.

### Baseline

Before the implementation of routine testing an audit was completed at two of four sites (Vilnius University Hospital and Victor Babes Hospital in Romania) to establish a baseline test rate. The audits included retrospective testing data from a period of 4–12 months prior to pilot initiation. For the pilots in Barcelona and the RPLC in Lithuania testing of chemsex-users and individuals with alcohol dependency was a new initiative and baseline test rates thus not available.

### Pilot

During the pilot period from July 2018 to December 2019, consecutive individuals attending any of the four sites were offered the appropriate suite of tests. Data on HIV, HCV-, HBV- and STI-testing was collected prospectively and reported manually in a secure online browser-based platform or extracted into excel. Three sites used the platform to report the following data: number of individuals seen with an IC, number tested and number of individuals with positive tests results. The RPLC in Lithuania reported data in excel on the number of visits by individuals in treatment for alcohol dependency, number of HIV and HCV tests conducted, and number of positive tests results per month. All data was reported fully anonymised and in aggregated form.

Ethical approvals were not required as testing for HIV and/or HBV and HCV was already part of standard care and integrated into the operational policy of the departments at all pilot sites. All testing of HIV, HBV, HCV and STI, was offered routinely as part of the medical investigation for the identified IC, consent to the testing was obtained verbally or in writing as legally required in each country.

### Combinations of integrated testing

The pilot sites implemented different combinations of integrated testing. The Vilnius University Hospital in Lithuania, started out by offering HIV tests to all individuals presenting with one of the following ICs: seborrheic dermatitis, candidiasis, psoriasis, herpes zoster and herpes simplex and STIs, after 10 months of implementation, HCV testing was added to the offer to all individuals presenting with one of the ICs; HBV testing was also added the last 6 months of the pilot period to individuals presenting with an STI. At Victor Babes in Romania, individuals presenting with TB were offered an HIV test.

Finally, the risk behaviours alcohol dependency and chemsex were added to the ICT strategy as indicators for testing. People treated for alcohol dependency were included into the existing testing programme and offered integrated HIV/HCV testing. Chemsex-users presenting at one of the facilities in the referral network, were referred for integrated HIV, HCV and STI testing.

### Tools and implementation

To facilitate pilot implementation of IC testing, training of staff and frequent staff meetings were conducted in the clinics before the pilots. In the Vilnius University Hospital, plan-do-study-act cycles (PDSA) were performed. PDSA provides a framework to implement and test changes on a small scale, in a structured way and build on the learnings and act immediately [12]. Test rates for all the ICs were calculated and shared at the staff meetings to motivate staff and keep the awareness level high. A list of ICs was displayed in all consultation rooms in the clinic as a visual reminder to the consultants. At Victor Babes a patient information leaflet (PIL) (please see Additional file 1) explaining the benefits of HIV testing in individuals with TB was introduced. The PIL was approved by the Ethics Committee at the hospital. It was introduced at staff meetings and was distributed to in-patients as well as out-patient by the nurses in the TB clinic. Health care workers at all the referral facilities in Barcelona received training in indications of chemsex and clinical suspicion of STIs before routine referral for HIV/HCV and STI testing was implemented. In the RPLC in Lithuania three staff training sessions on HIV and HCV testing and linkage to care were performed [13]. The pilot sites were encouraged to report any kinds of barriers encountered during implementation.

## Results

### Characteristics of participants

At the Vilnius University hospital, 3.664 consecutive individuals aged 18–65 years participated: 1.592 individuals with an STI, and 2.072 individuals with other dermatological ICs (Table 1). At Victor Babes Hospital, a total of 260 patients between the age of 18–65 years receiving treatment and care for TB, were offered an HIV test. All patients > 18 years of age undergoing treatment for alcohol dependency at the RPLC with unknown HIV and/or HCV status were offered testing for one or both infections. The majority of patients tested, were middle aged men of who had never previously tested for HIV nor HCV.

**Table 1:** Number of individuals tested, testing rates and positivity

Indicator condition	Baseline audit HIV test rate (Individuals tested/ seen)	Pilot Individuals seen	Pilot Individuals tested for HIV	Pilot Individuals testing positive	Pilot HIV test rate	Pilot HIV positivity	Baseline audit HCV test rate (Individuals tested/ seen)	Pilot Individuals tested for HCV	Pilot Individuals testing positive	Pilot HCV positivity	Pilot HCV test rate	Pilot Individuals tested for HBV	Pilot Individuals testing positive	Pilot HBV positivity	Pilot Individuals tested for STIs	Pilot Individuals testing positive	Pilot STI positivity
Established IC HIV prevalence of > 0.1%																	
STI	37.4% (375/1002)	1643	1592	1	97%	0,06%	0.8% (9/1104)	921	5	0.54%	98%	452	2	0.44%			
Herpes simplex (labialis)	0% (0/55)	105	45	0	43%	0%	NA	19	0		95%						
Herpes zoster	0% (0/48)	44	27	1	61%	3.70%	NA	14	0		93%						
Seborrheic dermatitis	2.2% (5/223)	357	206	1	58%	0,49%	NA	91	0		58%						
Severe psoriasis	0.5% (10/2090)	2534	1523	0	60%	0%	NA	567	3	0.53%	95%						
Candidiasis (oral)	3.3% (12/360)	478	271	0	57%	0%	NA	89	0		57%						
All dermatovener rological IC	10.6%	5161	3664	3	71%	0.08%	NA	1701	8	0.47%	NA						
Tuberculosis	67.4% (295/438)	308	260	2	84%	0,77%											
*Total established IC	NA	5469	3924	5	72%	0.13%											
New ICs included																	
Chemsex	NA	170	9	0	***5%	0	NA	117	4	3.4%	69%				117	61	52%
Alcohol dependency	NA	NA**	946	3	NA	0,3%	NA	926	34	3.67%	NA						

^*^All dermatovenerological IC and Tuberculosis

**Individuals seen is not available, individuals attending treatment are reported as visits and one individual has several visits during treatment

***Most chemsex-users where known HIV positive

Finally, chemsex-users referred for integrated testing at the HIV clinic were predominantly Men who have Sex with Men (MSM) who had been involved in chemsex between January 2018 and December 2019. Most of the participants in this pilot were already known HIV positive.

### Pilot phase testing rates

**Fig. 1:**
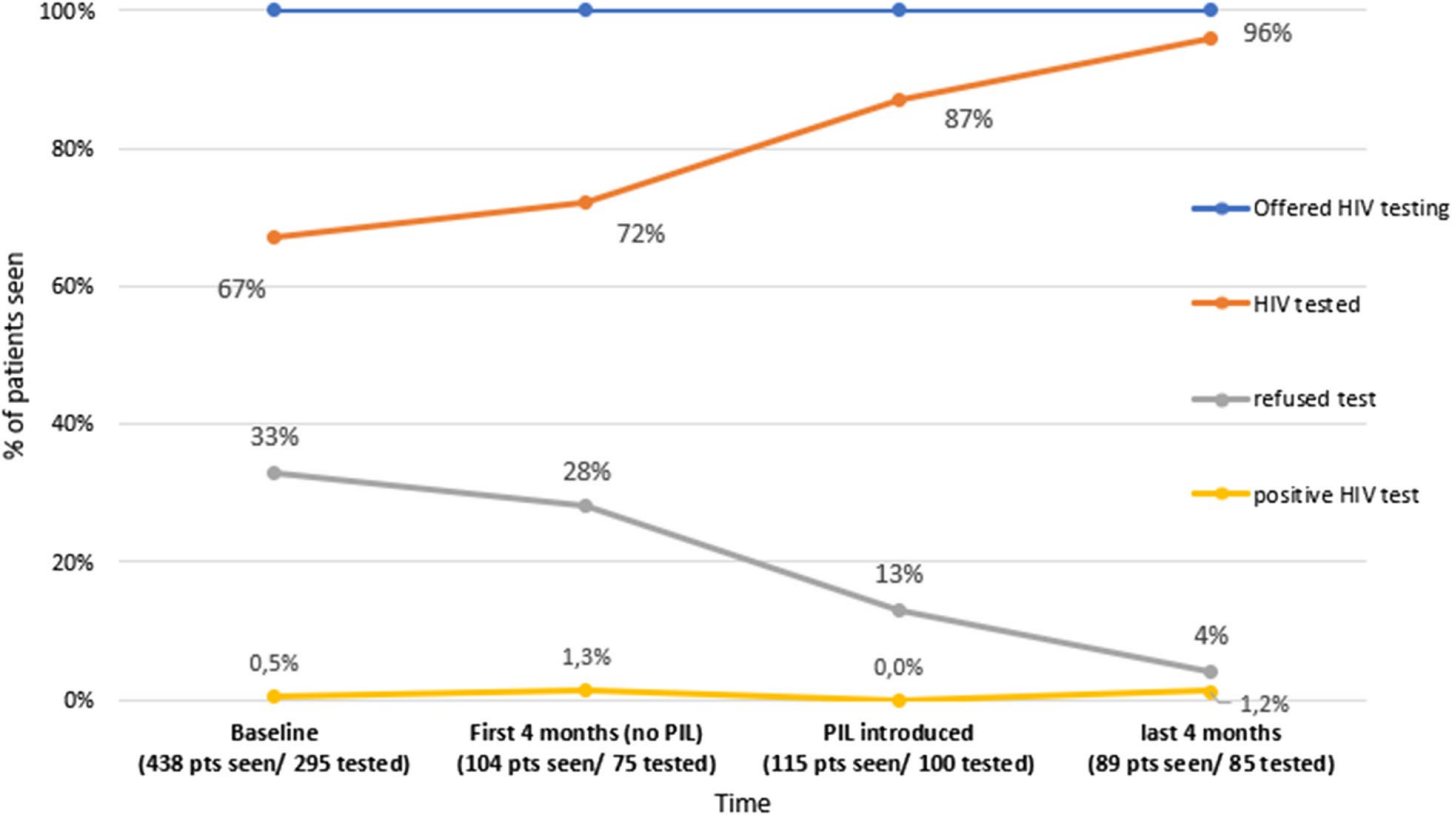
Increasing HIV testing in a TB clinic in Craoiva, Romania

During the 18-month pilot, the overall HIV testing rate for all IC^2^ at the Vilnius University Hospital increased from a baseline of 10.6% (range 0—37% for herpes simplex and STI respectively) to 71% (range 43—97% for herpes simplex and STI respectively) (Table 1). For HCV baseline testing rates for the dermatological ICs were unknown. The overall pilot HCV testing rate for all IC at the was 83% (range 57–98% for herpes simplex and STIs respectively) over a period of 11 months. For STI individuals with HCV testing increased from 0,8 to 98% and HBV testing increased from 0.6 to 92% over a period of 6 months (Table 1). At Victor Babes, 260 patients with TB were tested for HIV and the testing rates for TB increased from baseline 67 to 94% (Fig. 1) over a period of 8 months after the introduction of the PIL (see Additional file 1). At the RPLC the pilot period was 10 months and in that period the centre had a total of 4343 visits du to alcohol dependency (one individual has more than one visit). During the pilot period 946 HIV and 926 HCV tests were conducted in individuals seen with alcohol dependency (Table 1).

Over a period of 12 months 170 people were referred for testing at the HIV clinic in Barcelona, based on chemsex indications, for HIV/HCV and STI testing, of these 117 accepted testing for STIs and HCV (69%). Due to most of the referrals being known HIV positives, the HIV test rates were low and only nine people were tested for HIV.

### Positivity rates

Table 1 shows the number of individuals testing positive and the positivity rates according to number of people tested. At the Vilnius University Hospital three new cases of HIV (0.08% positivity) were identified during the pilot; one with Herpes zoster (3.7% positivity), one with Seborrheic dermatitis (0.5% positivity) and one with an STI (0.06% positivity)—all cases were linked to care. A total of eight new diagnosis of active HCV (0.47% positivity) were identified: five with an STI (0.54% positivity) and three with severe psoriasis (0.53% positivity). Two new cases of HBV were identified in individuals with an STI (0.44% positivity). In the 946 HIV tests performed in people with alcohol dependency, three new cases of HIV were identified (0.3% positivity) and 34 cases of HCV among 926 tests (3.7% positivity) were identified during the 10 months pilot period. At Victor Babes Hospital two new cases of HIV were identified among the 260 conducted tests (0.8% positivity). In chemsex-users the positivity of asymptomatic and undiagnosed STI was high at 52%, four people tested positive for HCV (3%) and zero tested positive for HIV.

All HIV, HCV and HBV tests performed were antibody tests and all individuals with a positive test were referred for further investigation and linked to treatment and care in an infectious disease hospital.

## Discussion

ICT is a proven, effective and cost-effective strategy for increasing HIV testing in health care facilities and is recommended in European and many national testing guidelines. In this study, the tools previously developed to support implementation of ICT for HIV were adapted to include routine offer of HCV, HBV and STI testing and the risk behaviours chemsex and alcohol dependency were added as indicators for routine offering of integrated testing. Overall, the pilot activities in this study have shown positive outcomes in terms of increased testing rates.

It is a limitation to the study that direct comparison of overall results across the pilots was not possible, because the pilot sites implemented different combinations of integrated testing. Furthermore, data was not reported consistently for all pilots and the pilot periods varied. The encountered barriers were multiple ranging from provider to structural and financial ones. The pilot sites implementing IC guided HIV and HCV testing experienced resistance among the staff to increase routine testing in certain indicators—either due to the extra workload or because they did not see the need for it. This was expressed at staff meetings and in reluctance to perform testing. Especially for the dermatological conditions where the number of individuals requiring blood samples is usually small and the nursing staff dedicated for this purpose limited; during the study monthly sampling rates increased up to ten-fold, which led to incapacity issues. Some physicians expressed reluctance to request testing for patients with dermatological conditions as they did not consider it to be relevant. This was overcome six to 10 months into the pilot, where the results led to a change of attitude among staff members, and the majority of dermatovenerology specialists at the Vilnius University Hospital have expressed that they now recognize i.e. seborrheic dermatitis, herpes zoster infections and psoriasis as relevant ICs for HIV and HCV testing. This was the result of staff trainings, persistence of pilot staff to remind their colleagues and the results obtained. Similarly, staff at the drug treatment centres did not consider individuals treated for alcohol dependency as a risk group and felt it was an extra and unwarranted task to offer testing to this patient group. However, the high positivity rates, especially for HCV, revealed a gap and a missed opportunity for integrated testing. It is most likely that some of these individuals are having a polysubstance abuse including use of intravenous drugs or an undisclosed history of intravenous drug use. In Romania written consent for an HIV test continues to be a legal requirement. Implementing the PIL was an attempt to overcome a structural barrier by preparing the patient and minimize the time spent by the clinician informing the patient and witnessing the consent in an already busy consultation. The intervention succeeded in increasing the HIV test coverage and proved to be a simple, efficient and low-cost tool to implement.

The sustainability of the increased testing rates is likely to depend on the staff supporting the interventions and the feasibility of the procedures. Hence continued training of staff, presentations and feedback of the results, as well as extra resources for tests and staff time will be required to avoid testing rates dropping to pre-pilot levels. The sustainability of the increased testing is of course also linked to financial costs. At Vilnius University Hospital, the financial cost for the increased testing was co-financed in collaboration with an NGO and a pharmaceutical company. At the RPLC, the relatively expensive rapid HCV tests were provided by an NGO but are not routinely available outside the pilot, hence it will be difficult to continue to offer the HCV testing for all clients—including those with alcohol dependency.

Finally, within the pilots testing data had to be collected and reported. At Vilnius University Hospital the preparation of monthly reports was time consuming as not all data was available electronically and most patient case records had to be checked manually. However, reporting testing data is also key to motivating staff and to demonstrate that the intervention is effective.

The method and tools used for implementing HIV ICT in Vilnius University Hospital: regular staff training and meetings, progress monitoring and PDSA cycles, have proved to be transferrable and effective methods for implementing integrated HIV/HCV testing for dermatology ICs and integrated HIV/HCV/HBV testing for STIs. Another positive outcome of this pilot has been a change of attitude among staff communicated from the site, bringing STI testing and screening in the Vilnius University Hospital in accordance with main European guidelines.

Introducing a PIL, a simple tool providing information to the patient on HIV testing and TB and saving time in the consultation, has proven an effective tool to increase HIV testing and acceptance rates in the TB dept in Victor Babes Hospital. Before the PIL was introduced, individuals with TB were likely to decline an HIV test or were simply not offered a test, due to lack of time. The PIL has been instrumental in overcoming a barrier, such as signed informed consent, to HIV testing. The successful implementation and acceptation of the PIL could be used as argumentation for removing the signed consent entirely.

HCV testing for people with alcohol dependency, showed high positivity rates, this could be due to unknown previous or current use of injectable drugs which identifies a gap in HIV and HCV testing and makes alcohol abuse a very relevant indicator condition for integrated HIV/HCV testing. Even if high positivity rates for STI among chemsex-users is not surprising, it underlines the importance of early testing for this group. The pilot showed chemsex-users present in other facilities than the established health care facilities, thus awareness of integrated testing of this group at the referral facilities is important for earlier diagnosis of especially STI, making chemsex an important indicator condition.

## Conclusions

The four pilot studies demonstrated that the ICT strategy can successfully be applied and adapted to increase testing rates for HIV/HCV/HBV/STI in health care settings.

The tools previously developed to increase routine HIV test offer in health care settings have proven feasible and effective to support the inclusion of additional indicators and routine testing for other infectious diseases.

In this study the ICT strategy has successfully been expanded to include alcohol dependency and chemsex as indicators for integrated HIV/HCV and STI testing. HCV testing of people with alcohol dependency in particular showed high positivity rates and identified a missed opportunity for testing HCV and HIV. Similar testing approaches should be widely adopted in other European health care settings.

## Supplementary Information

**Additional file 1.** Patient Information Leaflet.pdf on HIV testing for TB patients, distributed in the TB clinic in Romania.

## Data Availability

All data was collected aggregated and will be available on request.

## Abbreviations

HBV: Hepatitis B Virus
HCV: Hepatitis C Virus
HIV: Human Immunodeficiency Virus
IC: Indicator condition
ICT: Indicator condition guided HIV testing
MSM: Men who have Sex with Men
OST: Opiode substitution therapy
PDSA: Plan-do-study-act
PIL: Patient information leaflet
PWID: People with intravenous drug-use
RPLC: Republican Centre for Addictive Disorders
TB: Tuberculosis

## Footnotes

1 Dermatovenerological indicator conditions tested were: STI, herpes simplex virus, herpes zoster, seborrheic dermatitis, severe psoriasis, oral candidiasis.

2 STI, herpes simplex, herpes zoster, seborrheic dermatitis, severe psoriasis, oral candidiasis.
