# Supplementary material for: Increased integrated testing for HIV, hepatitis C and sexually transmitted infections in health care facilities: results from the INTEGRATE Joint Action pilots in Lithuania, Romania and Spain
Source: BMC Infect Dis. 2021 Sep 13;21(Suppl 2):845. doi: 10.1186/s12879-021-06537-2 (PMC8438813; doi:10.1186/s12879-021-06537-2)
Supplement: Supplementary file 1 — Additional file 1. Patient Information Leaflet.pdf on HIV testing for TB patients, distributed in the TB clinic in Romania. [file 12879_2021_6537_MOESM1_ESM.pdf]

HAVING AN HIV TEST AT THE TUBERCULOSIS CLINIC

Spitalul Clinic de Boli Infecțioase și Pneumoftiziologie “Victor Babeş” Craiova
Clinica Pneumoftiziologie I

Tuberculosis

You have been referred to this department du to suspected or confirmed infection with Tuberculosis (TB). TB is a contagious infection that usually affects the lungs, but it can also affect other parts of the body, like the spine or the brain. It is caused by bacteria and is transmitted via saliva – coughing and sharing household with a TB infected person. You can have the bacteria in your body, but your immune system stops them from spreading and you will neither be sick nor contagious. If your immune system is suddenly impaired, the bacteria will start spreading and you will get sick. One reason for an impaired immune system could be an HIV infection.

It is important to emphasize that most people with TB will NOT have HIV infection. But we think it is worthwhile that everyone takes the test.

A routine HIV test of all patients will help us to identify the patients with HIV and provide timely treatment and care

HIV (the Human Immunodeficiency Virus) is now *a manageable condition with medication. Treatment is very successful, but it is most successful if the infection is identified at an early stage. The majority of patients will be on straightforward treatment experiencing few, if any, side effects.*

As a patient at the Pneumophtisiology clinic you will be offered an HIV test. A blood test will be drawn together with your routine blood tests. You can ask any questions you may have, and you do not have to have the test. Declining to have an HIV test will in no way affect the care you receive.

You will be asked to sign a written consent to have the HIV test done.

Anyone who accepts an HIV test will receive the test result. The result is confidential and known only to you and your treating physician/nurse.
Taking the test, and testing negative, has no implications for insurance or mortgage applications.

If the result is positive, you will be referred to the infectious diseases ward, where you will receive treatment and care for HIV.

Thank you for taking the time to read this information.

Please keep this leaflet for your reference.

Date attended: _ _ / _ _ / _ __

Result due by: _ _ / _ _ / _ _ _

EFECTUAREA UNUI TEST HIV LA DISPENSARUL TBC

Spitalul Clinic de Boli Infecțioase și Pneumoftiziologie „Victor Babeş” Craiova
Clinica Pneumoftiziologie I

Tuberculoză

Ați fost direcționat către acest departament datorită suspiciunii sau confirmării infecției cu tuberculoză (TB). Tuberculoza este o infecție contagioasă care, de obicei, afectează plămânii, dar poate afecta și alte părți ale corpului, precum coloana vertebrală sau creierul. Este cauzată de o bacterie transmisă prin intermediul salivei – prin tuse și prin împărțirea spațiului de locuit cu o persoană infectată cu TB. Puteți trăi cu bacteria în corp, însă sistemul imunitar o va opri din a se răspândi și, în acest fel, nu veți fi niciodată bolnav sau contagios. Dacă sistemul dumneavoastră imunitar este slăbit brusc, atunci bacteria se va răspândi și vă veți îmbolnăvi. Unul dintre motivele pentru un sistem imunitar slăbit este infecția cu virusul HIV.

Este important a se sublinia faptul că majoritatea persoanelor cu tuberculoză NU vor avea niciodată infecție cu HIV. Însă noi suntem de părere că este de folos ca toată lumea să efectueze acest test.

Testarea HIV de rutină a tuturor pacienților ne va ajuta să îi identificăm pe cei care au virusul și să le oferim din timp tratament și îngrijire medicală

HIV (Virusul Imunodeficienței Umane) reprezintă acum *o boală care se poate menține sub control cu ajutorul medicației. Tratamentul are foarte mare succes, însă are cel mai mult succes atunci când infecția este identificată în fază incipientă. Majorității pacienților li se va administra tratamentul direct și vor experimenta câteva efecte secundare, poate chiar deloc.*

Ca și pacient al clinicii Pneumoftiziologie vi se va oferi un test HIV. Pe lângă proba de sânge uzuală, vi se va recolta o probă de sânge pentru acest test. Puteți să adresați orice întrebări ați putea avea. Refuzul efectuării unui test HIV nu va afecta în niciun fel îngrijirea medicală pe care o primiți.

Pentru a putea face acest test, vi se va cere să semnați un formular de consimțământ.

Orice persoană care acceptă efectuarea testului HIV, va primi rezultatele acestuia. Rezultatul este confidențial și va fi cunoscut numai de dumneavoastră și de medicul curant/asistentă. Efectuarea testului și, mai apoi, un rezultat negativ nu au implicații asupra cererilor de asigurare sau ipotecă.

Dacă rezultatul este pozitiv, veți fi direcționat către secția de boli infecțioase, unde veți primi tratament și îngrijiri medicale pentru infecția cu HIV.

Vă mulțumim că ați ales să citiți aceste informații.

Vă rugăm să păstrați acest pliant ca sursă de referință.
